# Influence of Deficiency of Lime and Phosphoric Acid on the Bones

**Published:** 1872-11

**Authors:** 


*Influence of Deficiency of Lime and Phosphoric Acid on the Bones.*—The "*Lancet*" says that Dr. Weiske, continuing his former experiments on the effect of the withdrawal of certain constituents of the body from the food, has selected a goat, and has supplied it with food deficient in lime and phosphoric acid. He gives the results of his experiments in the *Zeitschrift fur Biologie,* just published. They are remarkable, and show that the withdrawal of these elements from the food proves injurious to the animal, and may, in fact, ultimately lead to death; but that no change in the composition of the bones can be shown to have occurred, and that the bones do not appear to be rendered thereby more friable.

